# The integrins of the urochordate *Ciona intestinalis *provide novel insights into the molecular evolution of the vertebrate integrin family

**DOI:** 10.1186/1471-2148-5-31

**Published:** 2005-05-13

**Authors:** Richard Ewan, Julie Huxley-Jones, A Paul Mould, Martin J Humphries, David L Robertson, Raymond P Boot-Handford

**Affiliations:** 1 Wellcome Trust Centre for Cell-Matrix Research, Faculty of Life Sciences, Michael Smith Building, The University of Manchester, Oxford Road, Manchester M13 9PT, UK; 2 Plant Molecular Science Group, Bower Building, University of Glasgow, G12 8QQ, UK

## Abstract

**Background:**

Integrins are a functionally significant family of metazoan cell surface adhesion receptors. The receptors are dimers composed of an alpha and a beta chain. Vertebrate genomes encode an expanded set of integrin alpha and beta chains in comparison with protostomes such as drosophila or the nematode worm. The publication of the genome of a basal chordate, *Ciona intestinalis*, provides a unique opportunity to gain further insight into how and when the expanded integrin supergene family found in vertebrates evolved.

**Results:**

The *Ciona *genome encodes eleven α and five β chain genes that are highly homologous to their vertebrate homologues. Eight of the α chains contain an A-domain that lacks the short alpha helical region present in the collagen-binding vertebrate alpha chains. Phylogenetic analyses indicate the eight A-domain containing α chains cluster to form an ascidian-specific clade that is related to but, distinct from, the vertebrate A-domain clade. Two *Ciona *α chains cluster in laminin-binding clade and the remaining chain clusters in the clade that binds the RGD tripeptide sequence. Of the five *Ciona *β chains, three form an ascidian-specific clade, one clusters in the vertebrate β1 clade and the remaining *Ciona *chain is the orthologue of the vertebrate β4 chain.

**Conclusion:**

The *Ciona *repertoire of integrin genes provides new insight into the basic set of these receptors available at the beginning of vertebrate evolution. The ascidian and vertebrate α chain A-domain clades originated from a common precursor but radiated separately in each lineage. It would appear that the acquisition of collagen binding capabilities occurred in the chordate lineage after the divergence of ascidians.

## Background

Integrins are cell surface adhesion receptors which mediate cell-extracellular matrix (ECM), cell-cell and cell-pathogen interactions. Integrin receptors are structurally elaborate and composed of non-covalently associated α and β subunits. Integrins have a large extracellular domain responsible for binding extracellular ligands, a transmembrane domain and, a relatively small intracellular domain that interacts with the cytoskeleton and intracellular signaling pathways. Integrins integrate information from the extracellular and cytoplasmic environments by transducing signals bidirectionally across the plasma membrane. Hence, the binding of a specific ECM ligand by an integrin may elicit the activation of intracellular signaling pathways, cytoskeletal reorganisations and changes in cell adhesion or migration; and conversely, alterations in the intracellular environment and signaling can result in the activation or inhibition of ligand binding by the extracellular domain of an integrin [1,2]. Consequently, integrins have fundamental roles in diverse physiological processes including: tissue morphogenesis and remodeling [3], immune and inflammatory responses [4], and regulation of cell growth, migration and differentiation [5].

Integrin ligands include ECM components such as laminins, fibronectin and collagens [2], cell surface intercellular adhesion molecules (ICAMs) and plasma proteins such as fibrinogen [4]. Since cell adhesion and the production of a collagen-based ECM are essential characteristics of metazoa, it is not surprising that integrins have been detected throughout the multicellular animal kingdom, from the simplest and most primitive phyla (sponge and cnidarians) [6] to higher vertebrates. In humans, 18 α and 8 β integrin subunits combine to form 24 functionally distinct heterodimers [2].

Within the complement of 24 human integrin receptors, distinct functional sub-divisions can be made on the basis of ligand specificity and tissue distribution. A previous study of integrin phylogeny [7] identified 5 α subunit clades with vertebrate chains occupying 4 of these, namely: laminin binding (α3, α6 and α7 – PS1 clade), RGD tri-peptide binding (αV, αIIb, α5 and α8 – PS2 clade), and 2 vertebrate-specific clusters consisting of a small clade comprising the α4 and α9 subunits and a large αA-domain containing clade including both collagen-binding (α1, α2, α10 and α11) and leukocyte-specific (αD, αE, αL, αM and αX) α subunits (I-DOM clade). The αA-domain is structurally homologous to the module identified originally in von Willebrand factor. The collagen-binding αA-domains contain an inserted α-helix which appears to contribute directly to ligand binding. α subunit integrin homologues from model invertebrates *C. elegans *and *D. melanogaster *clustered in the laminin clade (PS1) and the RGD binding (PS2) clade with remaining α chains forming a drosophila-specific PS3 clade [7].

The α chain αA-domain, shared by all members of the I-DOM clade, mediates ligand binding in a metal ion-dependant manner by way of a conserved, non-contiguous sequence termed the metal ion-dependent adhesion site (MIDAS) motif [8,9]. It is somewhat surprising that no examples of collagen-binding (i.e. αA-domain-containing) integrins have been found in protostomes since basement membrane and fibrillar collagens are essential components of some of the most primitive invertebrates such as the cnidarian, *Hydra vulgaris *[10,11]. The origin of the I-DOM clade remains to be fully determined although since the urochordate *Halocynthia roretzi *has at least one α integrin containing an αA-domain [12] the limited evidence available to date suggests that it may be a chordate invention. In contrast, all known β subunits have a conserved βA-domain (also known as the I-like domain) [13] that must therefore have been present in the prototypic metazoan β subunit.

Vertebrate β subunits have been resolved into three branches by phylogenetic analysis, with a majority of sequences falling into two well-supported clades [7]. The two clades, termed β1 (β1, β2 and β7) and β3 (β3, β5, β6 and β8) included seven of the eight known subunits. In the β1 clade, the β2 and β7 subunits are leukocyte-specific, whereas their common ancestor, the β1 subunit, forms heterodimers with 12 of the 18 α subunits. Clade β3 subunits are all specific to RGD ligand binding integrins. The β4 subunit was positioned separately to other vertebrate clades and contains a unique extended cytoplasmic domain (~1000 residues), which makes direct contact with intermediate filaments via type fibronectin III repeats [14]. Protostome β subunits from *C. elegans *and *D. melanogaster *were not found to cluster with vertebrate sequences. Similarly, early deuterostome sequences (sea urchin) formed lineage-specific clusters with poor resolution amongst all invertebrate clades. A recent study by Miyazawa and Nonaka (2004) presented a phylogeny of integrin β subunits including novel sequences from the urochordate *Halocynthia roretzi*. These subunits are expressed on ascidian phagocytic blood cells (hemocytes) but phylogenetic analyses positioned them distal from vertebrate leukocyte β integrins in an ascidian-specific clade [15].

The recent sequencing of the genome of the ascidian *Ciona intestinalis *[16], a urochordate and one of the closest invertebrate relatives of vertebrates, provides a unique opportunity to gain insight into the complete set of integrins available in chordates prior to the large-scale or genome duplication events that many believe were associated with the early stages of vertebrate evolution [17-20]. An early preliminary analysis identified candidate integrin genes in the *Ciona *genome [21]. Here we have identified and refined the sequences of 11 α and 5 β integrin subunits from the *Ciona *genome. Eight of the α chains contain an αA-domain (also known as an I-Domain) that lacks the collagen-binding α helix and these chains form an ascidian-specific clade related to, but distinct from, the vertebrate I-DOM clade. The remaining 3 α chains are predicted to bind laminins and RGD-containing motifs. Two *Ciona *β chains cluster with the vertebrate β1 clade and the remaining 3 form an ascidian-specific β clade. The majority of chains in ascidian-specific clades are expressed on cells in the blood and are likely to be involved in innate immunological processes. These novel data provide further insights into the mechanism of evolution of the vertebrate family of integrins, and specifically when and how specific clades of integrin chains first arose. The differences between the ascidian and vertebrate complements of integrins again emphasises how phyla and species mould their genomes by the amplification of specific subsets of genes as part of the process of acquiring a stable and successful phenotype.

## Results

Obtaining refined protein sequences for integrins encoded in the Ciona genome

**Figure 1:**
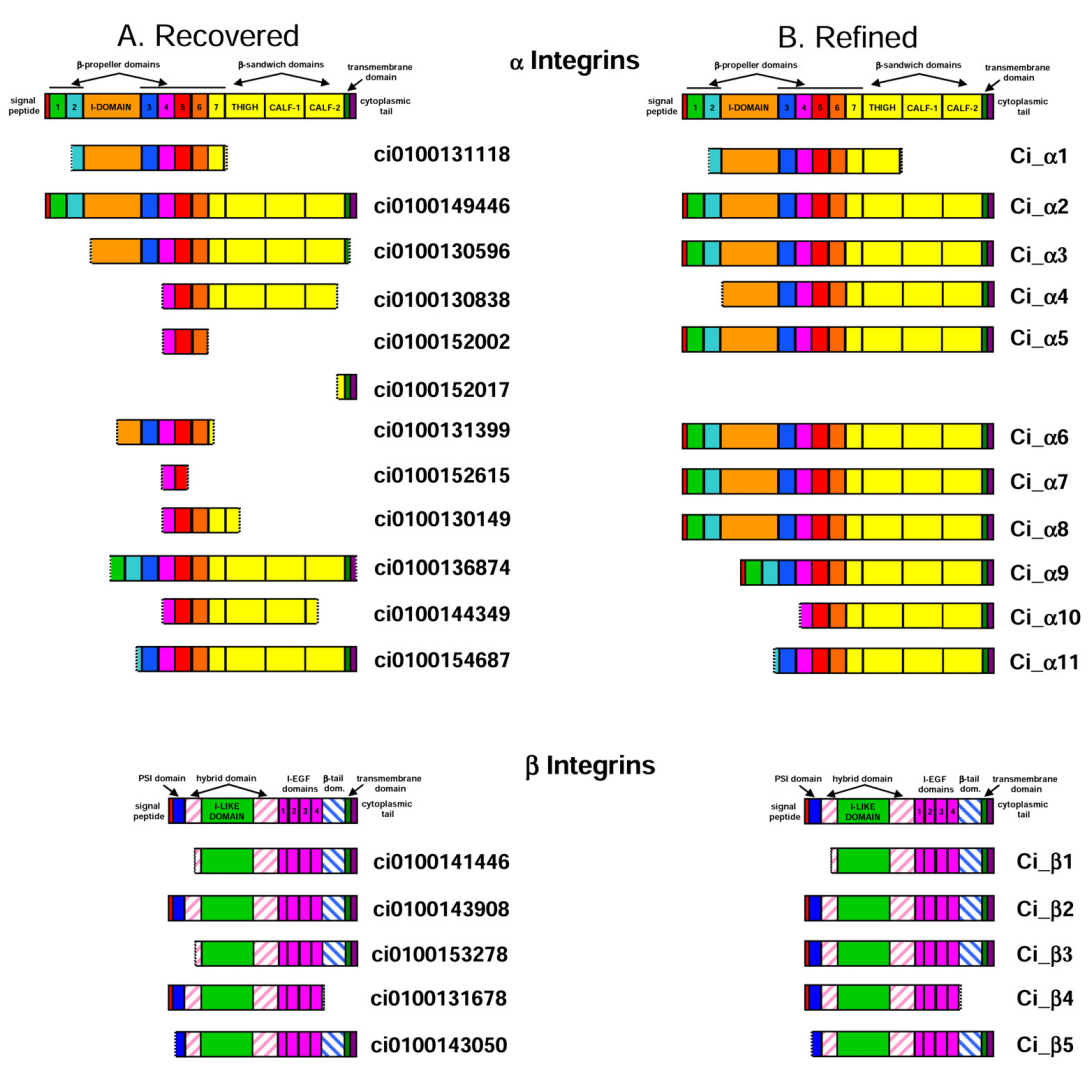
Comparison of sequences recovered directly from the JGI *C. intestinalis *database before (A – Recovered) and after (B – Refined) sequence refinement. The annotated cartoons represent the domain structures of generic α & β integrin chains. Each subsequent row represents: (A – Recovered) the domain structure encoded by a sequence as retrieved directly from the database together with its assigned database accession number; and (B – Refined) the refined version of that gene after detailed analysis of the genomic sequence as described in the methods together with the name assigned during these analyses (e.g. Ci_α1). Refined sequences are presented as alignments in Fig. 3-7 and are also included as amino acid residue sequence files (see additional file 1).

**Figure 2:**
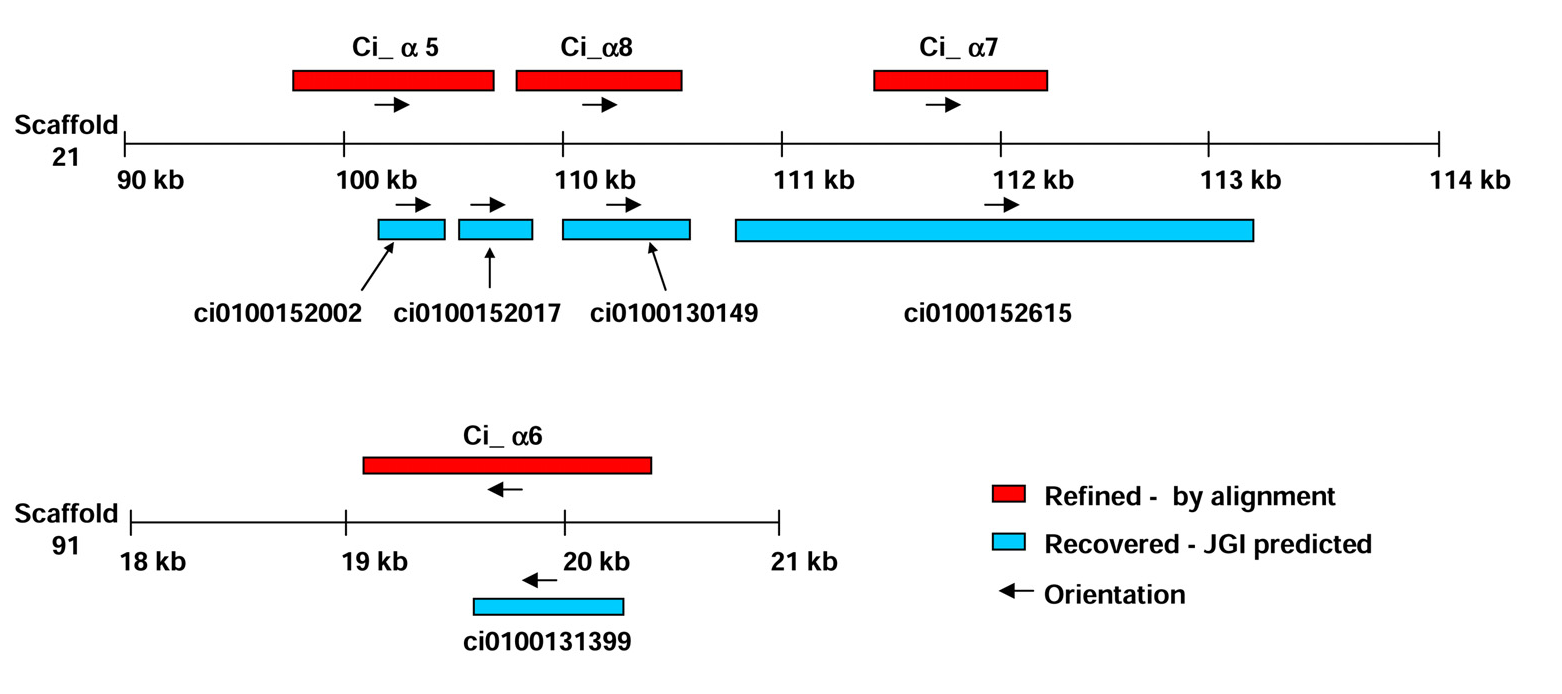
The genomic locations and orientations of recovered and refined α integrin genes present on scaffolds 21 and 91. The genomic locations and orientations of the four very closely related A-domain containing α integrin genes identified after sequence refinement (Ci-α5–8, coloured red) together with the original five JGI-predicted gene fragments (blue) are indicated.

The *C. intestinalis *genome database was searched for α and β integrin genes and the sequences listed in Fig. 1A were obtained. These sequences were already annotated and no additional genes were obtained during extensive BLAST searching of the *Ciona *genome using the 26 human integrin chain sequences. The 12 annotated α chain sequences recovered directly from genome database clearly represented fragments of genes, whereas the five β chain sequences represented a much more complete data set (Fig. 1A). In order to generate more complete α chain data, DNA flanking the predicted gene fragments were downloaded and searched manually for putative "missing" exons. The search involved the identification of ORFs based on their sequence being present in EST clones or conservation of their translated sequence in comparison with homologous genes. Eleven *Ciona *α chain genes were confirmed by this process (Fig. 1B) with two of the JGI predicted genes (ci0100152017 & ci0100152002 – Fig. 1A) proving to represent different regions of a single gene (Fig. 2). The sequence refinement process was aided by the fact that four of the *Ciona *α chain genes were very highly conserved and had clearly radiated relatively recently by a process involving tandem duplication. The completion of one full-length gene sequence therefore considerably simplified the elucidation of the exon structures of the remaining three genes since these were well conserved (Table 1), which was not the case when comparing the gene structures of more distantly related integrins (data not shown). The size and relative genomic loci of these four genes (ci100152017, ci0100130149, ci0100152615 & ci0100131399 – subsequently referred to as α5–8) is presented in Fig. 2.

**Table 1:** Exon sizes (bp) of Ci_α5–8 genes.

Scaffold	21	91	21	21
JGI acc no	ci0100152002	ci0100131399	ci0100152615	ci0100130149
Exon no	Ci_a5	Ci_a6	Ci_a7	Ci_a8

1	170	161	167	167
2	128	128	128	128
3	63	63	63	63
4	127	127	67	127
5	123	123	121	123
6	116	92	80	90
7	187	205	187	219
8	179	179	181	179
9	57	68	68	68
10	179	180	180	180
11	87	88	90	86
12	256	256	234	256
13	210	210	232	210
14	307	310	313	304
15	149	146	146	146
16	224	224	224	224
17	143	143	143	143
18	80	107	107	107
19	87	89	89	89
20	101	99	99	99
21	121	120	122	120
22	108	109	109	109
23	129	129	129	129
24	114	114	126	202
25	88	88	76	135
26	135	132	135	97
27	151	92	95	No Exon

### Sequence alignments and characterization

**Figure 3:**
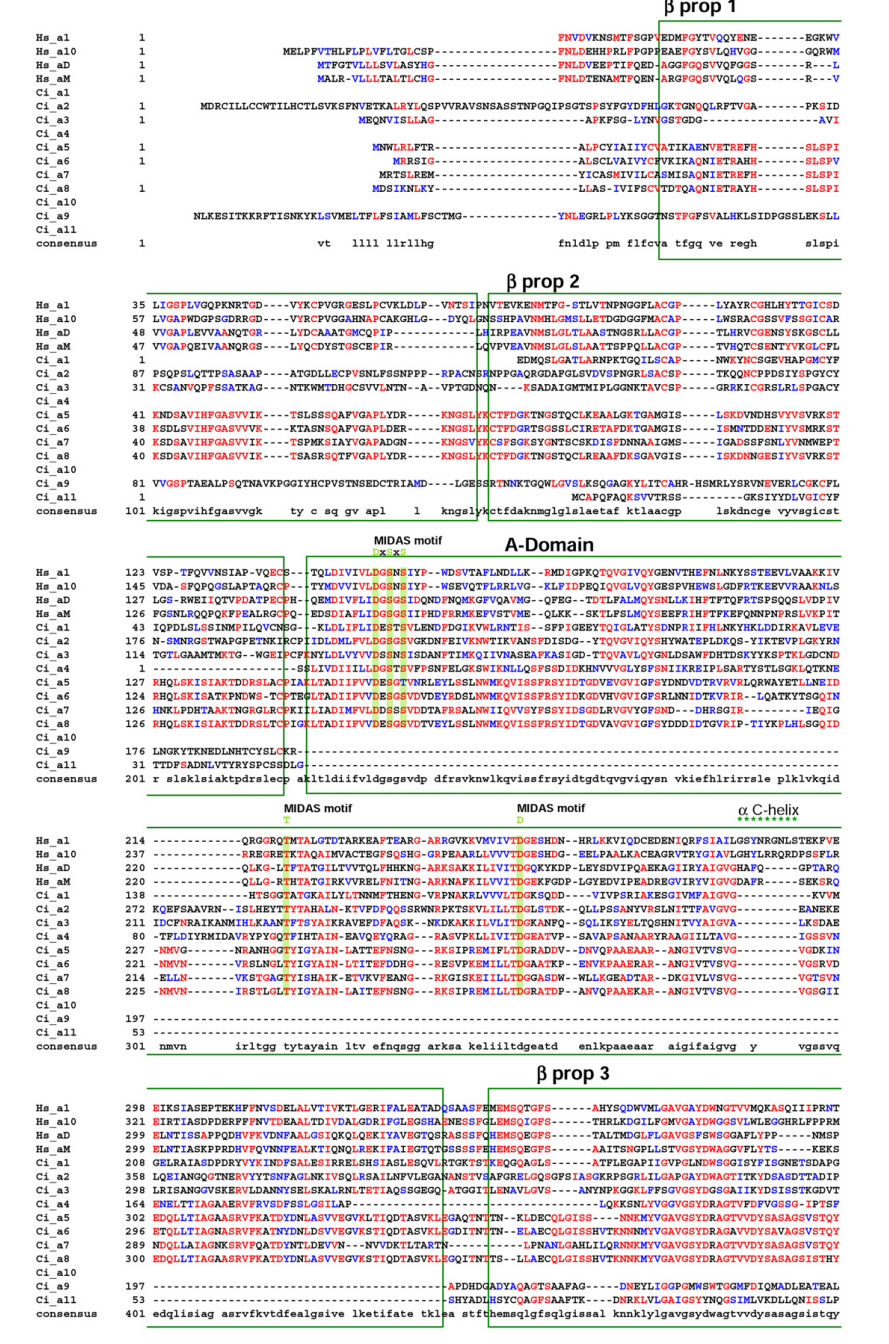
Alignment of the refined *Ciona *α chain sequences with representative human orthologues (residues 1-391 based on human α1 integrin chain). Protein domains and conserved motifs are annotated. Levels of sequence conservation are indicated (>50% identical, red; conservative substitutions, blue). MIDAS and α C-helix within the inserted A-domain are highlighted, as are the β-propeller domains 1–3.

**Figure 4:**
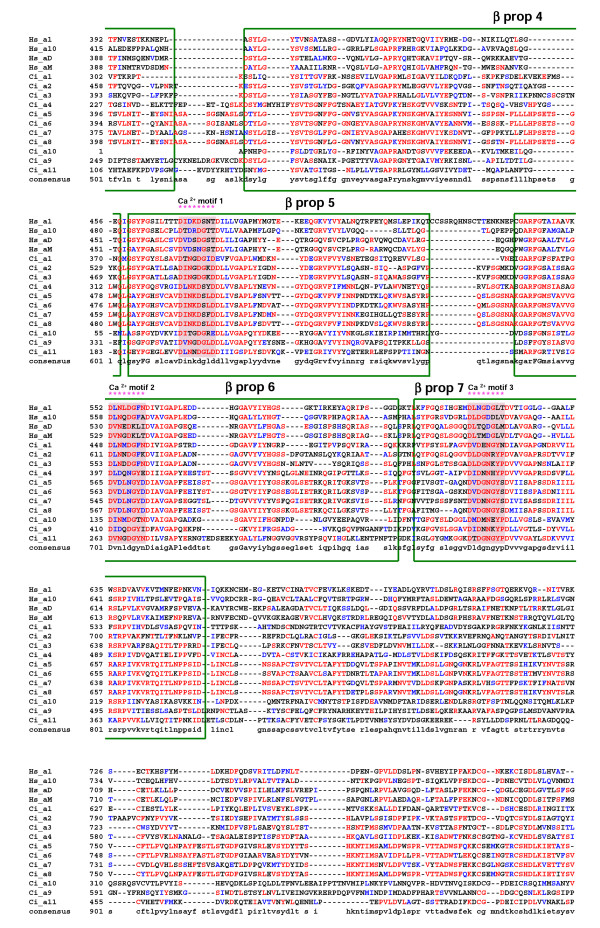
Alignment of the refined *Ciona *α chain sequences with representative human orthologues (residues 392-796 based on human α1 integrin chain). Protein domains and conserved motifs are annotated. Levels of sequence conservation are indicated (>50% identical, red; conservative substitutions, blue). Ca^2+^-binding motifs in β-propeller repeats 5–7 are highlighted.

**Figure 5:**
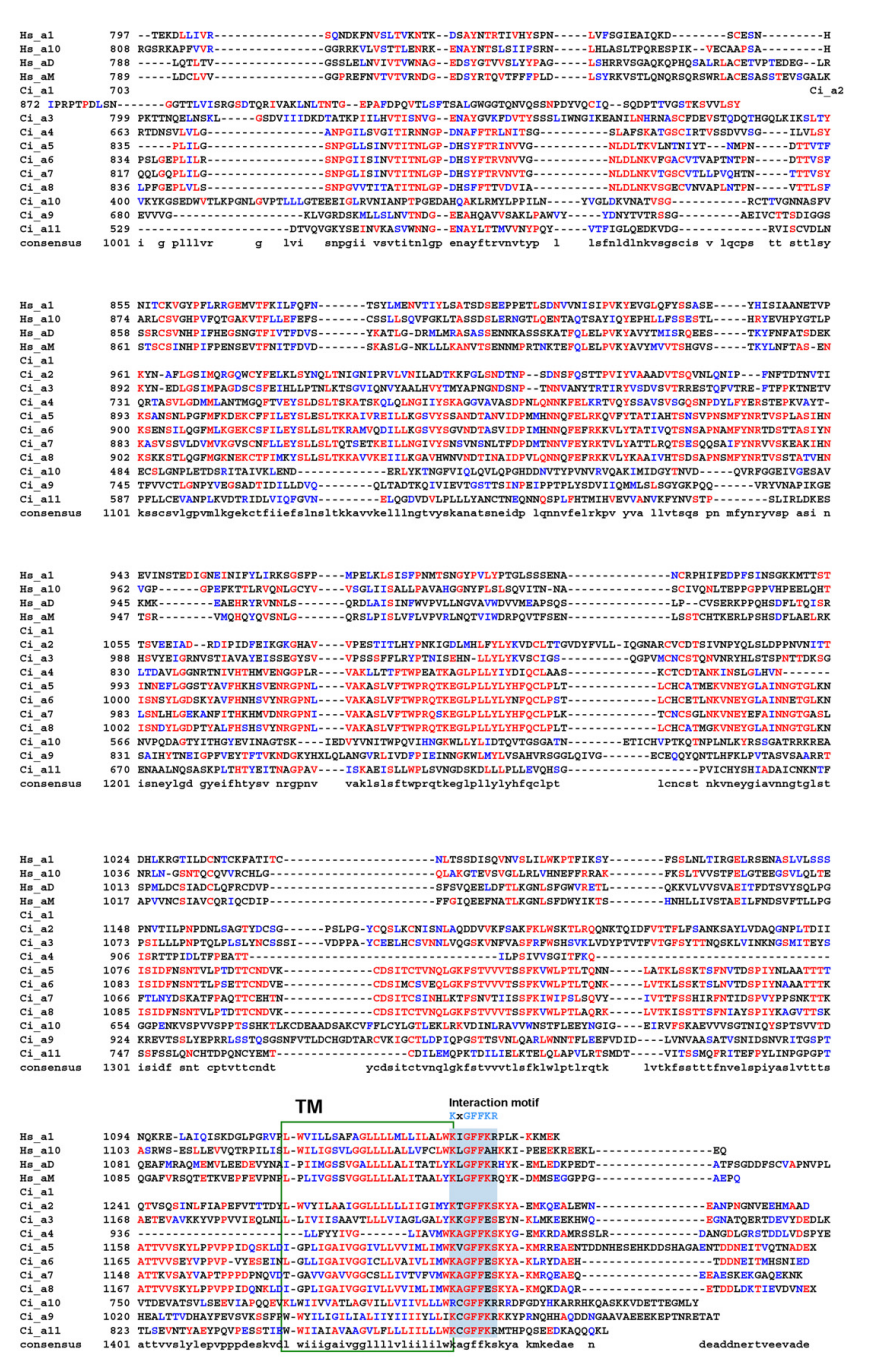
Alignment of the refined *Ciona *α chain sequences with representative human orthologues (residues 797 to C-terminus based on human α1 integrin chain). Protein domains and conserved motifs are annotated. Levels of sequence conservation are indicated (>50% identical, red; conservative substitutions, blue). Transmembrane domain (TM) and cytoplasmic interaction motif are indicated.

The sequences for the 11 *Ciona *α chains were aligned with human homologues. An annotated version of this alignment is presented in Fig. 3,4,5. Eight of the 11 *Ciona *α chains (α1–8) have a well conserved αA-domain including the essential residues that constitute the MIDAS motif (Fig. 3). The position of the αA-domain insertion in the human and *Ciona *chains is identical, indicating that all these chains have arisen from a common progenitor. However, all 8 *Ciona *chains lack 9–11 amino acid residues corresponding to the 'collagen binding' α-helical domain present in the collagen binding (Hs_α1 & α10) vertebrate α chains (Fig. 3). The *Ciona *α chains share all other major features with their vertebrate homologues across the alignment including the well-defined transmembrane and conserved intracellular interaction domains (Fig. 3,4,5).

**Figure 6:**
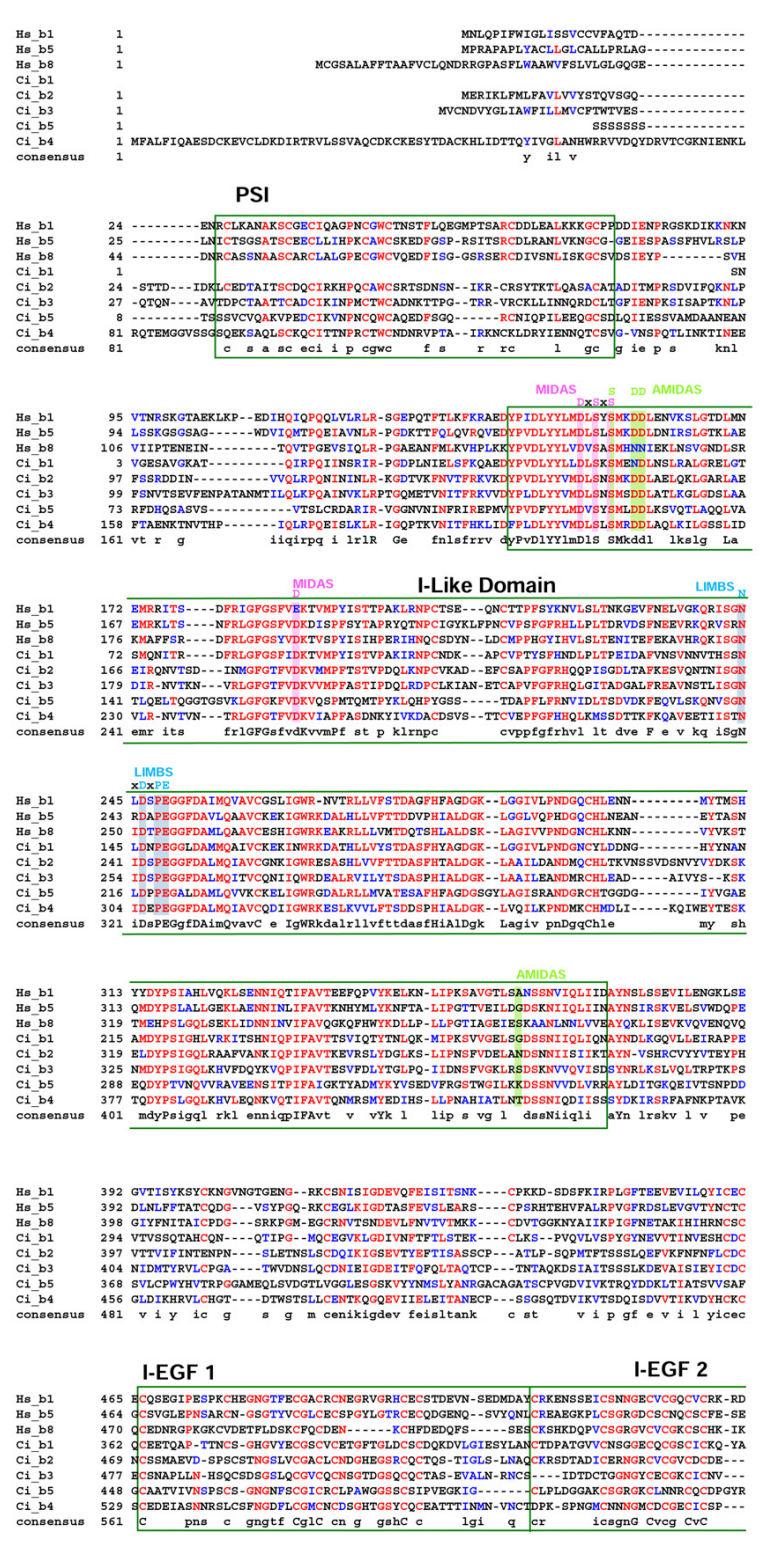
Alignment of the refined *Ciona *β chain sequences with representative human orthologues (residues 1-542 based on the human β1 integrin chain). Protein domains and conserved motifs are annotated. Levels of sequence conservation are indicated (>50% identical, red; conservative substitutions, blue). Adjacent to MIDAS (AMIDAS), ligand associated metal binding site (LIMBS) and MIDAS cation binding sites, and interaction motifs are highlighted as are the plexin/semaphorin/integrin (PSI), β-A domain (I-like) and epidermal growth factor (EGF) domains 1–2.

**Figure 7:**
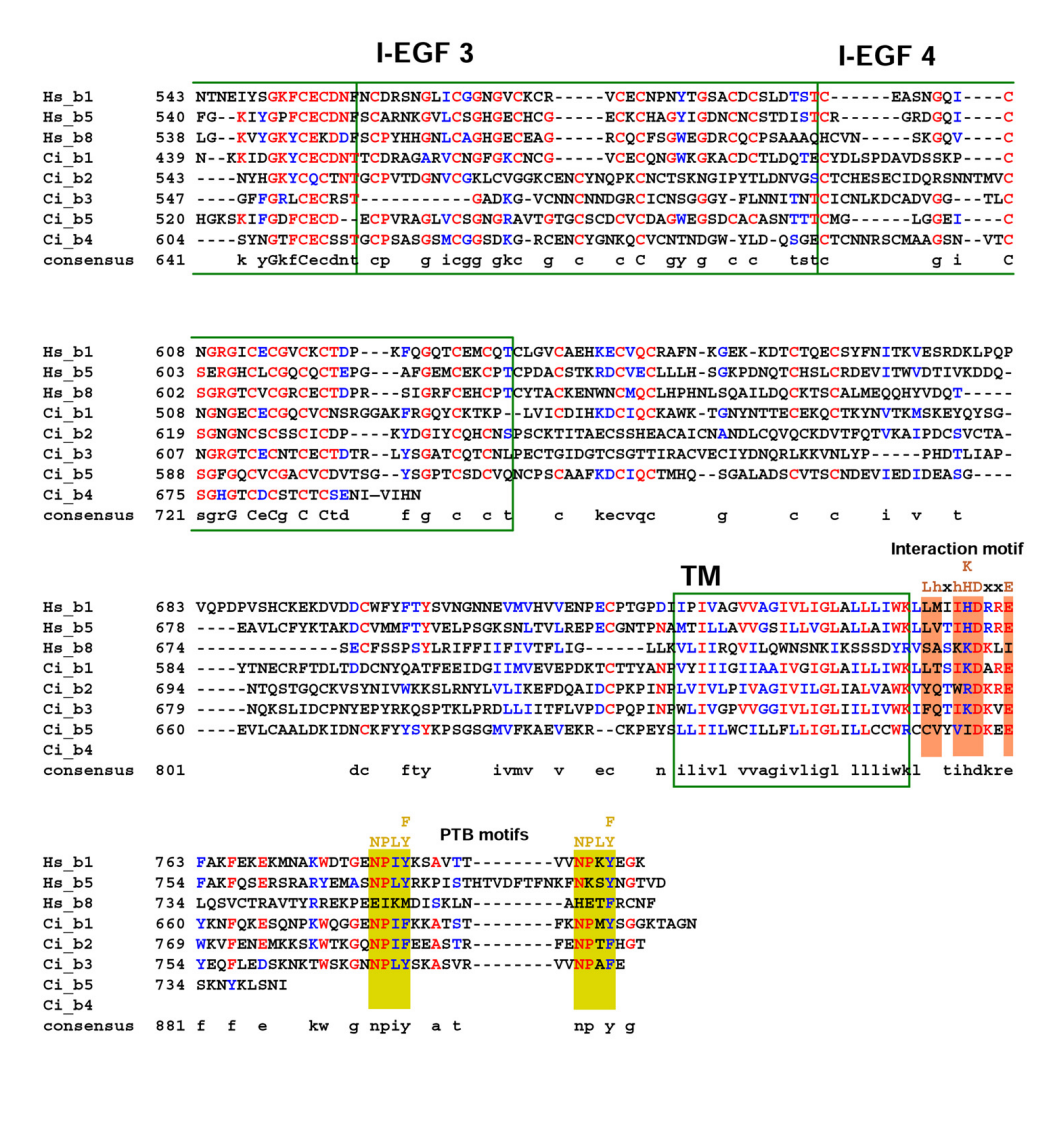
Alignment of the refined *Ciona *β chain sequences with representative human orthologues (residues 543 to the C-terminus based on the human β1 integrin chain). Protein domains and conserved motifs are annotated. Levels of sequence conservation are indicated (>50% identical, red; conservative substitutions, blue). EGF domains 2–4, transmembrane (TM) domain, interaction and phosphotyrosine binding (PTB) motifs are indicated.

The sequences of the five *Ciona *β chains are also highly conserved with respect to their vertebrate orthologues including the MIDAS motif within the I-like domain, the four EGF domains, the transmembrane domain, and the intracellular interaction motifs and PTB-like domains (Fig. 6 &7).

### Phylogenetic analyses

**Figure 8:**
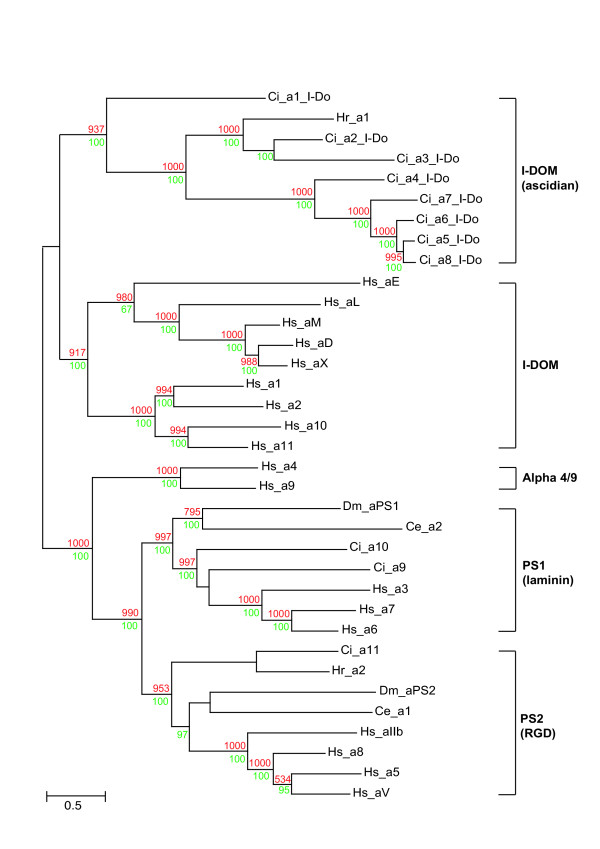
Phylogenetic relationship of *Ciona *α integrin chains with representative protostome and vertebrate orthologues. Maximum Likelihood tree is shown with supporting Neighbor Joining bootstrap replicates (red) and Bayesian clade credibility values (green). Horizontal scale is amino acid replacements per site.

The α chain analysis is presented in Fig. 8 in the form of a maximum likelihood tree with supporting data from 1,000 neighbor-joining bootstrap replicates and Bayesian analysis. Overall the inferred phylogenetic relationships are consistent with a previous phylogenetic reconstruction [7]. The clades identified by Hughes (PS1, PS2, and the vertebrate I-DOM and α4/9) are all present (Fig. 8). Note, the PS3 clade is not shown because it is specific to *Drosophila*. *Ciona *α9 and α10 cluster in the PS1 clade and their position, separating the protostome and vertebrate sequences, is as expected. In contrast, *Ciona *α11 clusters with its ascidian orthologue (Hr_α2) in the PS2 clade but at an anomalous position distal to the protostome sequences. Only Neighbor Joining analysis (not shown) produced the anticipated branching in this region of the tree with protostome PS2 clade members being most distal. The remaining eight *Ciona *α chains (Ci_α1 to α8) all have an αA-domain and form an ascidian specific clade that includes the αA-domain containing *H. roretzi *α1 chain (I-DOM [ascidian] – Fig. 8).

**Figure 9:**
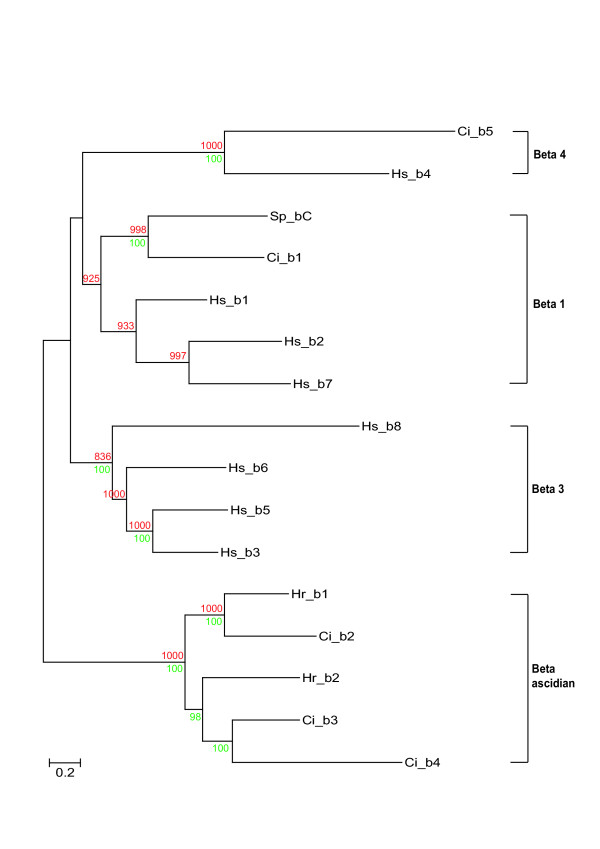
Phylogenetic relationship of *Ciona *β integrin chains with representative deuterostome orthologues. Maximum Likelihood tree is shown with supporting Neighbor Joining bootstrap replicates (red) and Bayesian clade credibility values (green). Horizontal scale is amino acid replacements per site.

The β chain analysis is presented in Fig. 9. Again, phylogenetic relationships are consistent with Hughes [7]. The vertebrate clades β1 and β4 include *Ciona *orthologues (Ci_β1 and β5 respectively). The vertebrate β3 clade has no identified *Ciona *orthologue (Fig. 9). The remaining 3 *Ciona *β chains (Ci_2 to 4) form an ascidian specific clade including *H. roretzi *β1 & 2 chains (Fig. 9).

## Discussion

The urochordate *C. intestinalis *occupies a pivotal position in the animal kingdom for understanding the evolution of vertebrates. The *Ciona *genome provides an insight into the basic set of genes available at the very beginning of vertebrate evolution since the urochordates diverged just prior to the widespread gene duplication processes that are thought to have shaped and transformed the vertebrate genome [16].

The *Ciona *genome encodes 11 α and five β integrin chains. As expected, some *Ciona *α chains cluster in the PS1 laminin-binding clade (Ci_α9 & α10), and in the PS2 RGD-binding clade (Ci_α11 together with its ascidian orthologue Hr_ α2 – see Fig 8). It is unclear why the urochordate members of the PS2 clade cluster in an anomalous position distal to the protostome sequences although this suggests that the ancestral urochordate PS2 gene underwent significant and rapid sequence changes after divergence from the lineage leading to vertebrates. The remaining eight *Ciona *α chains all contain an αA-domain and, somewhat surprisingly, form an ascidian-specific clade related to, but distinct from, the vertebrate I-DOM clade. This phylogenetic relationship suggests the vertebrate and ascidian αA-domain containing clades arose from a common progenitor but that this gene radiated separately in both the ascidian and vertebrate lineages after their divergence. Data supporting this hypothesis includes: i) ascidian and vertebrate genes have the αA-domain inserted at the same location supporting the notion of both lineages having a common progenitor (Fig. 3); ii) all eight ascidian α chain αA-domains lack the same 9–11 amino acids encompassing and adjacent to the α-helical domain in the collagen-binding α chains (Fig. 3); and iii) at least four of the *Ciona *αA-domain-containing α chains (α5–α8) appear to have arisen very recently as a result of tandem duplications within the ascidian genome based on retained similarities in exon size (Table 1), their high level of sequence identity (Fig. 3,4,5 and 8) and their genomic location (Fig. 2). The common progenitor gene presumably evolved in deuterostomes, possibly in the earliest chordates since the vertebrate and ascidian lineages of αA-domain α chains have radiated entirely separately and no protostome orthologues have been identified. The most likely function for the progenitor αA-domain-containing α chain involves the adhesion of blood cells to complement-like proteins or the extracellular matrix. In the urochordate *H. roretzi*, the Hr_α1 αA-domain gene, together with the Hr_β1 & β2 genes, are expressed on hemocytes and are thought to act as complement receptors [12]. A large number of EST's for Ciona αA-domain-containing α chains have been found in either blood cell or hemocyte cDNA libraries (Table 2). Finally, the expression of half of the genes comprising the vertebrate I-DOM clade is leukocyte-specific. In addition, it would seem that the collagen-binding property exhibited by the other half of the I-DOM α chains was a late functional acquisition of this vertebrate clade, perhaps associated with an insertional mutagenic event creating the collagen binding α-helix. It is noteworthy that *Ciona *expresses progenitor forms for all three clades of vertebrate fibrillar collagens [22]. It is therefore apparent that the early evolution of chordates did not require collagen-binding integrins. Functionally important interactions between integrins and collagen triple helices must have developed later in chordate evolution, possibly in the earliest vertebrates and co-incidental with the acquisition of the collagen-binding helix.

**Table 2:** Expression profiles of A-domain containing α-integrins in *Ciona intestinalis*. Data has been obtained from the TIGR Gene Indices database .

Ciona Integrin	JGI Acc Code	TIGR cDNA Index Acc Code	Tissue specific expression from EST data
Ci_a1	Ci0100131118	BW029582	Blood cells
Ci_a2	Ci0100149446	TC42900	Blood cells
Ci_a3	Ci0100130596	TC56905	Heart, neural complex, digestive gland
Ci_a4	Ci0100130838	TC66015	(Whole embryo only)
Ci_a5	Ci0100152002	TC6115, TC63051, TC63231	Blood cells, heart, hemocytes
Ci_a6	Ci0100131399	TC73566, TC69775	Blood cells, heart, neural complex
Ci_a7	Ci0100152615	TC59274	Blood cells, digestive gland, gonad
Ci_a8	Ci0100130149	TC75204	Blood cells, neural complex, gonad

The phylogenetic relationships of the *Ciona *and vertebrate β integrin chains (see Fig. 9) emphasizes the pivotal position invertebrate chordates occupy with respect to understanding how vertebrates and their genes evolved. Previous phylogenetic analysis has suggested that neither protostome nor early deuterostomes (echinoderm) β chain sequences cluster with their vertebrate orthologues [7]. The clustering of a *Ciona *(Ci_β1) and a previously reported echinoderm sequence (Sp_ βC) with the vertebrate β1 clade genes (Fig. 9) resolves more clearly how the promiscuous vertebrate β1 chain and its paralogues have evolved from a deuterostome-specific progenitor. In addition, the vertebrate β4 chain, which fails to cluster with any other vertebrate genes, has a *Ciona *orthologue (Ci_ β5, Fig. 9) and must therefore have evolved prior to the divergence of ascidians. It was not possible to determine for certain whether the Ci_ β5 chain has the extended intracellular C-terminal domain present in the vertebrate β4 chain using either EST analysis or the search for exons containing conserved ORFs. Nevertheless, direct translation of 10 kb of genomic sequence 3' to the predicted C-terminus of the *Ciona *gene revealed the presence of short ORFs homologous to a domain present in the vertebrate integrin β4 intracellular domain and shared by Na^+^-Ca^2+ ^exchangers (data not shown). The remaining three *Ciona *β gene sequences formed an ascidian-specific clade together with two β chains from *H. roretzi *(Fig. 9).

**Figure 10:**
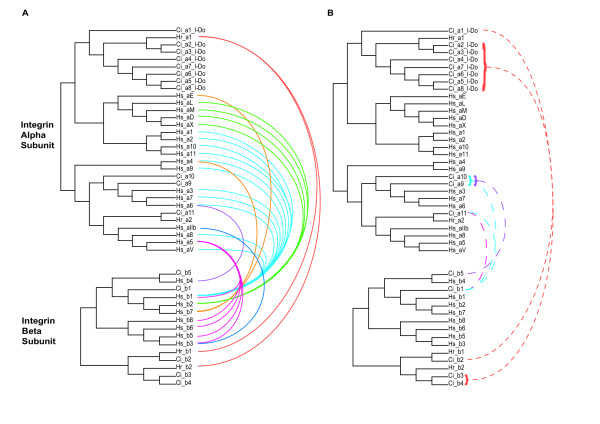
Prediction of dimerisation patterns for novel integrin chains based on a combination of known interactions and phylogeny. A. Schematic phylogenies of α and β chains with established heterodimer pairing indicated by adjoining solid colored lines. B. Heterodimer pairings predicted (dashed lines) on the basis of the data presented in A. The color coding in B related to the known parings in A used to make the prediction.

As phylogenetic relationships between novel α and β chains become defined, it is possible to start predicting likely interactions based on the known dimerisation partners of close relatives (Fig. 10). For instance, the vertebrate β1 chain dimerises with the laminin (PS1) and RGD (PS2) clade α chains. It is therefore probable that the *Ciona *β1 clade orthologue (Ci_β1) dimerises with the *Ciona *PS1 and PS2 clade α chains (Ci_α9, α10 & α11; see Fig. 10). Likewise, it has been established that the 2 β chains from *H. roretzi *(Hr_ β1 & β2) dimerise with Hr_α1 [15] and it is therefore probable that the related *Ciona *β2–4 chains partner *Ciona *α chains in the ascidian αA-domain clade (Ci_α1–8; Fig. 10). In *H. roretzi*, the β1, β2 and α1 chains are expressed on hemocytes [15] and it is noteworthy that the *Ciona *orthologues (Ci_α1–8 & β2–4) are also expressed predominantly in blood tissues based on EST analysis (data not shown).

The phylogenetic relationships of integrin genes within the vertebrate and invertebrate branches of the chordate phylum provides new insights into the evolution of both of these divergent lineages. The integrin gene complement of the ascidian genome gives a strong indication of the numbers and classes of integrin chains available to organisms at the very start of vertebrate evolution. For α integrins, it would appear that there was a minimum of one laminin binding (PS1), one RGD binding (PS2) and one αA-domain containing chain (Fig. 8). The novel *Ciona *data therefore clearly indicate that radiation of vertebrate α chain genes took place after the divergence of urochordates. The β chain phylogeny (Fig. 9) indicates that the common progenitor of urochordates and vertebrates had a minimum of one β1-like gene (that subsequently radiated in vertebrates) and a single Hs_β4/Ci_ β5 progenitor that radiated in neither lineage (Fig. 9). The origins of the ascidian and vertebrate-specific β clades is not resolved.

The ascidian lineage exhibits amplifications of subsets of integrin genes to produce ascidian-specific classes of novel integrins (Fig. 8,9,10). In particular, these novel integrins appear to be expressed in blood (see above) and may be involved in mechanisms associated with innate immunity. It has been proposed that the major metamorphic transformations between ascidian larval and adult body forms may be dependent upon innate immune responses [23]. This suggestion would provide a plausible explanation of the requirement for an expanded set of urochordate hemocyte integrins although more generic explanations, such as the preparation for a sessile life-style under attack by pathogens, are also possible.

## Methods

The complete sequences of the 26 known human integrin genes were used to probe the *Ciona intestinalis *genome and TIGR cDNA gene index ( and  using TBLASTN and PSI-BLAST with cut-off expectancy values of E = 1) to identify homologous genes [16,24]. *Ciona *gene models were also detected using the orthologue detection program InParanoid using a keyword search using 'integrin' as the query [25]. To identify all the integrin genes, reciprocal BLAST searches of the *Ciona*, human and non-redundant databases were used. Frequently, EST for the *Ciona *genes contradicted the proposed gene models from JGI. In instances where an EST clearly demonstrated the misplacement of exons in the recovered JGI model, the protein sequence was corrected to reflect this. To detect missing exons not supported by EST data, genomic DNA flanking the sequence of interest was retrieved and analysed using the GENESCAN [26] and GENEWISE [27] gene prediction programmes. Modified sequences were checked by aligning with respective human integrin profiles using CLUSTAL X [28] and corrected coding sequences used for subsequent analyses. Expression profiles for the *Ciona *genes were obtained from the TIGR database (see above).

The α and β integrin sequences were aligned separately using CLUSTAL X. The variable domain structure amongst α integrins necessitated subdivision of the alignment groups based on the presence/absence of an αA-domain. Subgroups were aligned and then combined so that the final alignment contained all the α integrins with a 200-residue (approx) gap region corresponding to the αA-domain.

For phylogenetic analysis, gap-containing sites were removed from each alignment and Maximum Likelihood trees were inferred using PROML from the PHYLIP package [29]. The JTT model of amino acid substitutions was used with and without global rearrangements and correction for rate heterogeneity (α value obtained from TREEPUZZLE [30]). The topologies of the trees were tested using two independent methods: Neighbour-joining bootstrap replicates and Bayesian tree inference using PHYLIP and Mr Bayes programmes respectively [31]. The accession numbers for protein sequences used in this study are presented in Tables 3 &4.

**Table 3:** Summary of α integrins used in phylogenetic analysis.

Lineage	Species	Database	Acc Code	Gene
Chordate	H. sapiens	Swiss-Prot	P56199	Hs_α1
Chordate	H. sapiens	Swiss-Prot	P17301	Hs_α2
Chordate	H. sapiens	Swiss-Prot	P08514	Hs_αIIb
Chordate	H. sapiens	Swiss-Prot	P26006	Hs_α3
Chordate	H. sapiens	Swiss-Prot	P13612	Hs_α4
Chordate	H. sapiens	Swiss-Prot	P08648	Hs_α5
Chordate	H. sapiens	Swiss-Prot	P23229	Hs_α6
Chordate	H. sapiens	Swiss-Prot	Q13683	Hs_α7
Chordate	H. sapiens	Swiss-Prot	P53708	Hs_α8
Chordate	H. sapiens	Swiss-Prot	Q13797	Hs_α9
Chordate	H. sapiens	Swiss-Prot	O75578	Hs_α10
Chordate	H. sapiens	Swiss-Prot	Q9UKX5	Hs_α11
Chordate	H. sapiens	Swiss-Prot	Q13349	Hs_αD
Chordate	H. sapiens	Swiss-Prot	P38570	Hs_αE
Chordate	H. sapiens	Swiss-Prot	P20701	Hs_αL
Chordate	H. sapiens	Swiss-Prot	P11215	Hs_αM
Chordate	H. sapiens	Swiss-Prot	P06756	Hs_αV
Chordate	H. sapiens	Swiss-Prot	P20702	Hs_αX
Urochordate	C. intestinalis	JGI Ci v1.0	See Fig. S1	Ci_α1
Urochordate	C. intestinalis	JGI Ci v1.0	ci0100149446	Ci_α2
Urochordate	C. intestinalis	JGI Ci v1.0	See Fig. S1	Ci_α3
Urochordate	C. intestinalis	JGI Ci v1.0	See Fig. S1	Ci_α4
Urochordate	C. intestinalis	JGI Ci v1.0	See Fig. S1	Ci_α5
Urochordate	C. intestinalis	JGI Ci v1.0	See Fig. S1	Ci_α6
Urochordate	C. intestinalis	JGI Ci v1.0	See Fig. S1	Ci_α7
Urochordate	C. intestinalis	JGI Ci v1.0	See Fig. S1	Ci_α8
Urochordate	C. intestinalis	JGI Ci v1.0	See Fig. S1	Ci_α9
Urochordate	C. intestinalis	JGI Ci v1.0	See Fig. S1	Ci_α10
Urochordate	C. intestinalis	JGI Ci v1.0	ci0100154687	Ci_α11
Urochordate	H. roretzi	Genbank	AB048261	αHr1
Urochordate	H. roretzi	Genbank	AB048262	αHr2
Arthropod	D. melanogaster	Swiss-Prot	Q24247	Dm_aPS1
Arthropod	D. melanogaster	Swiss-Prot	P12080	Dm_aPS2
Arthropod	D. melanogaster	Swiss-Prot	U76605	Dm_aPS3
Arthropod	D. melanogaster	Swiss-Prot	AAF58154	Dm_a16827
Arthropod	D. melanogaster	Swiss-Prot	AAF47029	Dm_a5372
Nematode	C. elegans	Swiss-Prot	P34446	Ce_a1
Nematode	C. elegans	Swiss-Prot	Q03600	Ce_a2
Echinoderm	S. purpuratus	Swiss-Prot	AF177914	Sp_aP
Porifera	G. cydonium	Swiss-Prot	X97283	Gc_alpha

**Table 4:** Summary of β integrins used in phylogenetic analysis

Lineage	Species	Database	Acc code	Gene
Chordate	H. sapiens	Swiss-Prot	P05556	Hs_b1
Chordate	H. sapiens	Swiss-Prot	P05107	Hs_b2
Chordate	H. sapiens	Swiss-Prot	P05106	Hs_b3
Chordate	H. sapiens	Swiss-Prot	P16144	Hs_b4
Chordate	H. sapiens	Swiss-Prot	P18084	Hs_b5
Chordate	H. sapiens	Swiss-Prot	P18564	Hs_b6
Chordate	H. sapiens	Swiss-Prot	P26010	Hs_b7
Chordate	H. sapiens	Swiss-Prot	P26012	Hs_b8
Urochordate	C. intestinalis	JGI Ci v1.0	ci0100141446	Ci_b1
Urochordate	C. intestinalis	JGI Ci v1.0	ci0100143908	Ci_b2
Urochordate	C. intestinalis	JGI Ci v1.0	See Fig. S1	Ci_b3
Urochordate	C. intestinalis	JGI Ci v1.0	ci0100131678	Ci_b4
Urochordate	C. intestinalis	JGI Ci v1.0	ci0100143050	Ci_b5
Urochordate	H. roretzi	Genbank	AB154831	Hr_b1
Urochordate	H. roretzi	Genbank	AB154832	Hr_b2
Echinoderm	S. purpuratus	NCBI	AF0559607	Sp_bC
Echinoderm	S. purpuratus	NCBI	NP_999732	Sp_bG
Echinoderm	S. purpuratus	NCBI	NP_999731	Sp_bL
Arthropod	D. melanogaster	Swiss-Prot	P11584	Dm_bPS
Arthropod	D. melanogaster	Swiss-Prot	L13305	Dm_bv
Nematode	C. elegans	Swiss-Prot	Q27874	Ce_b-pat3
Mollusc	B. glabraba	Swiss-Prot	AF060203	Bg_beta
Cnidaria	A. millepora	Swiss-Prot	AF005356	Am_beta
Porifera	G. cydonium	Swiss-Prot	O97189	Gc_beta

## Authors' contributions

RE and JHJ performed the database searches and analyses. RBH, DLR and MJH conceived of the study. All authors participated in the interpretation of data and in the writing of the manuscript.

## Supplementary Material

Additional File 1 Amino acid sequences of the refined *Ciona intestinalis *alpha and beta integrin chains (see Fig. 1) used to produce alignments in Figs 3,4,5,6,7 inclusive. Click here for file
