# Supplementary material for: The integrins of the urochordate Ciona intestinalis provide novel insights into the molecular evolution of the vertebrate integrin family
Source: BMC Evol Biol. 2005 May 13;5:31. doi: 10.1186/1471-2148-5-31 (PMC1145181; doi:10.1186/1471-2148-5-31)
Supplement: Additional File 1 — Amino acid sequences of the refined Ciona intestinalis alpha and beta integrin chains (see Fig. 1) used to produce alignments in Figs 3,4,5,6,7 inclusive. [file 1471-2148-5-31-S1.doc]

Additional file 1. Amino acid sequences of the refined *Ciona intestinalis* alpha and beta integrin chains used to produce alignments in Figs 3-7 inclusive

Ci_a1 (Refined from ci0100131118)

EDMQSLGATLARNPKTGQILSCAPNWKYNCSGEVHAPGMCYFIQPDLSLSSINMPILQVCNSGKLDLIFLIDESTSVLENDFDGIKVWLRNTISSFPIGEEYTQIGLATYSDNPRIIFHLNKYHKLDDIRKAVLEVEHTSGGTATGKAILYLTNNMFTHENGVRPNAKRLVVVLTDGKSQDDVIVPSRIAKESGIVMFAIGVGKVVMGELRAIASDPDRYVYKINDFSALESIRRELSHSIASLESQVLRTGKTSTKEQGQAGLSATFLEGAPIIGVPGLNDWSGGISYFISGNETSDAPGVFTKRPTKSSLIQYSITTGVFRKNSSEVILVGAPRMLSIGAVYILDKQDFLSKPKFSDELKVEKEFMSNQMGSYFGYSLSAVDTNGDGIDEVFVGAPLWMDKNYDEGRVFVYVSNETEGSITQREVVLSGVNEIGARFGFSFATPGDLNMDGFNDIVIGAPSTRDGDGAVFIYNGRPEGITPVPSQVIRASQKKKRPVYFSYSLFAGVDVDENGYNDIAVGAPGNDRVVILFSRPVIHVDLSVSASPQVINTTTPSKAHTNDSCNDNGTRTCVTVKACFHAYGVSTPEAIILRYQFEADVDYSGAKPRGRFNKYKGNLKIISNTTECIESTLYLKLPIYKQLEPLIVSVEYKLSPKMTVSKKSALLDIFDANQARTEVTFTKVCSDSHCESDLRINGIITX

Ci_a2 (Refined from ci0100149446)

MDRCILLCCWTILHCTLSVKSFNVETKALRYLQSPVVRAVSNSASSTNPGQIPSGTSPSYFGYDFHLGKTGNQQLRFTVGAPKSIDPSQPSLQTTPSASAAPATGDLLECPVSNLFSSNPPPRPACNSRNPPGAQRGDAFGLSVDVSPNGRLSACSPTKQQNCPPDSIYSPGYCYNSMNRGSTWAPGPETNKIRCPIIDLDMLFVLDGSGSVGKDNFEIVKNWTIKVANSFDISDGYTQVGVIQYSHYWATEPLDKQSYIKTEVPLGKYRNKQEFSAAVRNISLHEYTTYTAHALNKTVFDFQQSSRWNRPKTSKVLILLTDGLSTDKQLLPSSANYVRSLNITTFAVGVGEANEKELQEIANGQGTNERVYYTSNFAGLNKIVSQLRSAILNFVLEGANANSTVSAFGRELGQSGFSIASGKRPSGRLILGAPGAYDWAGTITKYDSASDTTADIPTFTQVQGVLPNRTKESYLGYSVSTGLYDGSGKQFVASGAPRYMLEGGVVLYEPQVGSSNFTNSQTIQAYGSYKQLGSYFGATLLSADINGDGKDDLLVGAPLYIGDNYDEGRVFVYLSQASNSIQASPGFVPKVFSGMKDVGGRFGSAISSAGDLNNDGFKDVIIGAPLADNGAGVVYVYHGSSDFGTANSLQYKQRIAATALSGTNLQYFGQSLQGGVDLDGNRYPDVAVGAPRSDTVVIFRTRPVAKFNTTLTFNKLNVPIFECFRREFRDCLQLRACIGLSGKGLEKSIKTLFSVVLDSSVTKKRVEFRNQANQTANGYTSRDIVLNITTPAAPVCFNYPVYVKTSIEDYSEPIVATMTYSLSSSHLAVPLSSISDPFIPKVKTASTSFETDCGVDQTCSYDLSIAGTQYIIPRPTPDLSNGGTTLVISRGSDTQRIVAKLNLTNTGEPAFDPQVTLSFTSALGWGGTQNVQSSNPDYVQCIQSQDPTTVGSTKSVVLSYKYNAFLGSIMQRGQWCYFELKLSYNQLTNIGNIPRVLVNILADTKKFGLSNDTNPSDNSFQSTTPVIYVAAADVTSQVNLQNIPFNFTDTNVTITSVEEIADRDIPIDFEIKGKGHAVVPESTITLHYPNKIGDLMHLFYLYKVDCLTTGVDYFVLLIQGNARCVCDTSIVNPYQLSLDPPNVNITTPNVTILPNPDNLSAGTYDCSGPSLPGYCQSLKCNISNLAQDDVVKFSAKFKLWSKTLRQQNKTQIDFVTTFLFSANKSAYLVDAQGNPLTDIIQTVSQSINLFIAPEFVTTTDYLWVYILAAIGGLLLLLLIIGIMYKTGFFKSKYAEMKQEALEWNEANPNGNVEEHMAAD

Ci_a3 (Refined from ci0100130596)

MEQNVISLLAGAPKFSGLYNVGSTGDGAVIKCSANVQPFSSATKAGNTKWMTDHGCSVVLNTNAVPTGDNQNKSADAIGMTMIPLGGNKTAVCSPGRRKICGRSLRLSPGACYTGTLGAAMTMKTGWGEIPCFKNYLDLVYVVDSSNSISDANFTIMKQIIVNASEAFKASIGDTTQVAVLQYGNLDSAWFDHTDSKYYKSPTKLGDCNDIDCFNRAIKANMIHLKAANTFTSYAIKRAVEFDFAQSKNKDKAKKILVLITDGKANFQSQLIKSYELTQSHNITVYAIGVALKSDAELRISANGGVSKERVLDANNYSELSKALRNLTETIAQSSGEGQATGGITLENAVLGVSANYNPKGGKLFFSGVGSYDGSGAIIKYDSISSTKGDVTSHKQVPGLFPKFPKDSYLGYSIASGYFEGNGTLYVATGAPRHALLGLVLVYEPQTDNSVSNPRIIKPNNCSSCSTNYKQLGSYFGATLLSADINGDGKDDLLVGAPLYIGDNYDEGRVFVYLSQASNSIQAWASSGFVPKVFSGMKDVGGRFGSAISSAGDLNDDGFNDVIIGAPLADNGAGVVYVYHGSNNLNTVYSQRIQSSSLQFPHLSNFGLSTSSGADLDGNKYPDVVVGAPNSNLAIIFRSRPVARFSAQITLTPPRRDIFECLQLPSRKCFNVTSCLTVYGKSVEDFLDVNVMILLDKSKKRLNLGGFNNATKEVKLSRNVTSCWSYDVYVTKNMIDFVSPLSAEVQYSLTSTHSNTPMSSMVDPAATNKVSTTASFNTGCTSDLFCSYDLMVNSSITLPKTTNQELNSKLGSDVIIIDKDTATKPIILHVTISNVGENAYGVKFDVTYSSSLIWNGIKEANILNHRNASCFDEVSTQDQTHGQLKIKSLTYKYNEDLGSIMPAGDSCSFEIHLLPTNLKTSGVIQNVYAALHVYTMYAPNGNDSNPTNNVANYTRTIRYVSDVSVTRRESTQFVTREFTFPKTNETVHSVYEIGRNVSTIAVAYEISSEGYSVVPSSSFFLRYPTNISEHNLLYLYKVSCIGSQGPVMCNCSTQNVNRYHLSTSPNTTDKSGPSILLLPNPTQLPLSLYNCSSSIVDPPAYCEELHCSVNNLVQGSKVNFVASFRFWSHSVKLVDYPTVTFVTGFSYTTNQSKLVINKNGSMITEYSAETEVAVKKYVPPVVIEQLNLLLIVIISAAVTLLLVIAGLGALYKKGFFESEYNKLMKEEKHWQEGNATQERTDEVYDEDLKQ

Ci_a4 (Refined from ci0100130838)

SSLIVDIIILLDGSTSVFPSNFELGKSWIKNLLQSFSSDIDKHNVVVGLYSFSNIIKREIPLSARTYSTLSGKLQTKNETFLDIYRMIDAVRYPYGQTFIHTAINEAVQEYQRAGRASVPKLLIVITDGEATVPSAVAPSANAARYRAAGIILTAVGIGSSVNENELTTIAGAAERVFRVSDFSSLGSILAPLQKKSNLYVGGVGSYDRAGTVFDFVGSSGIPTSFTGSINVDELKTTFEPETIQSLKDSYMGMYHIFYSVTSGNFFGTSNAEYIATGVPKYHSKGTVVVSKSNTPITSQSQVHSVHPYGSLWQLGSYFGQSVRGIDLNKDSYDDLVVSAPLYNEVNGYDQGRVFIFMNNLQNPVLAWVNETYQPRVLSGTKASGARFGMSIASAGDLDQNGYEDIIIGAPYEESTSTSSGVVYVYYNSQLGLNEINRQGIPGTTLKSSIQQFGYSVLGGTAIPVDIDNNHYPDVVVGAPRSDSVFLLKSRPIVDVQATIELIPPVFDVINCLADVTACSTVKICIKAKFRREHAPATLGMDLSTVLDSKIFDSQSKRITFFGKTTSVETKTLSVTSTYTCFVYSVKLNANALGTSAGALEISPTISFSYDFTAAHKQAVLSGIIDPLKEKKISADWTFNKSCSGTNGKCVHDLSVSATYSIRTDNSVLVLGANPGILSVGITIRNNGPDNAFFTRLNITSGSLAFSKATGSCIRTVSSDVVSGILVLSYQRTASVLGDMMLANTMGQFTVEYSLDSLTSKATSKQLQLNGIIYSKAGGVAVASDPNLQNNKFELKRTVQYSSAVSVSGQSNPDYLFYERSTEPKVAYTLTDAVLGGNRTNIVHTHMVENGGPLRVAKLLTTFTWPEATKAGLPLLYIYDIQCLAASKCTCDTANKINSLGLHVNISRTTPIDLTFPEATTILPSIVVSGITFKQLLFYYIVGLIAVMWKAGFFKSKYGEMKRDAMRSSLRDANGDLGRSTDDLVDSPYELAIN

Ci_a5 (Refined from ci0100152002 and ci0100152017)

MNWLRLFTRALPCYIAIIYCVATIKAENVETREFHSLSPIKNDSAVIHFGASVVIKTSLSSSQAFVGAPLYDRKNGSLYKCTFDGKTNGSTQCLKEAALGKTGAMGISLSKDVNDHSVYVSVRKSTRHQLSKISIAKTDDRSLACPIAKLTADIIFVVDESGTVNRLEYLSSLNWMKQVISSFRSYIDTGDVEVGVIGFSYDNDVDTRVRVRLQRWAYETLLNEIDNMVGNRANHGGTYIGYAINLATTEFNSNGRKSIPREMIFLTDGRADDVDNVQPAAEAARANGIVTVSVGVGDKINEDQLLTIAGAASRVFKATDYDNLASVVEGVKLTIQDTASVKLEGAQTNTTNKLDECQLGISSNNKMYVGAVGSYDRAGTVVDYSASAGSVSTQYTSVLNITEYSNIASASGSNASLSDTYLGYSVTSGNFFGGNVSYVASGAPKYNSKGMVAIYESNNVISSPSNFLLLHPSETSGLWQLGAYFGHSVCAVDINKDSLDDLIVSAPLFSNVTTGYDQGKVFVFINNPNDTKLQKWVSASYRPQSLSGSNAKGARFGMSVAVVGDVDLNGYDDIAVGAPFEEISSTGSGAVYIYYGSSKGLSETRKQRITGKSVTSPLKTFGGFIVSGTGAQNDVDGNGYSDVIISAPSSDRIIILRARPIVKVRTQITLNPPSIDLINCLSNSSAPCTSVTVCLTAFYTDDQVLTSPAPINVTMKLDSLLGNQGNKRLVFAGTTSSIHIKVYNVTSSRVCFTLPVQLNPAYFESTLSTGDFGIVSRLEVSYDYTTSHKNTIMSAMLDPWSPRVTTADWSFQKKCSEMKGTRCSHDLKIETAYSPLILGSNPGLLSINVTITNLGPDHSYFTRINVVGNLDLTKVLNTNIYTNMPNDTTVTFKSANSNLPGFMFKDEKCFFILEYSLESLTKKAIVREILLKGSVYSSANDTANVIDPMMHNNQFELRKQVFYTATIAHTSNSVPNSMFYNRTVSPLASIHNINNEFLGGSTYAVFHKHSVENRGPNLVAKASLVFTWPRQTKEGLPLLYLYHFQCLPLTLCHCATMEKVNEYGLAINNGTGLKNISIDFNSNTVLPTDTTCNDVKCDSITCTVNQLGKFSTVVVTSSFKVWLPTLTQNNLATKLSSKTSFNVTDSPIYNLAATTTTATTVVSKYLPPVPPIDQSKLDIGPLIGAIVGGIVLLVVIMLIMWKVGFFKSKYAKMRREAENTDDNHESEHKDDSHAGAENTDDNEITVQTNADEX

Ci_a6 (Refined from ci0100131399)

MRRSIGALSCLVAIVYCFVKIKAQNIETRAHHSLSPVKSDLSVIHFGASVVVKKTASNSQAFVGAPLDERKNGSLYKCTFDGRTSGSSLCIRETAFDKTGAMGISISMNTDDENIYVSMRKSTRHQLSKISATKPNDWSTCPTEGLTADIIFVVDESGSVDVDEYRDSLNWMKQVISSFRSYIDKGDVHVGVIGFSRLNNIDTKVRIRLQATKYTSGQINNMVNVRSLNGLTYIGYAINLTITEFDDHGRESVPKEMILLTDGAATKPENVKPAAERARANGIVTVSVGVGSRVDETQLLTIAGNASRVFKATNYDNLDSVVEGVKSTIQDTASVKLEGDITNTTNELAECQLGISSHVTKNNNMYVGAVGSYDRAGAVVDYSAVAGSVSTQYRSVLNITQYANIASASGISANLSDTYLGYSVTSGNFFGGNGEYVASGAPRYNSKGMVAVYEANNVMESSSKFLLLHPSETSGLWQLGAYFGHSVCAVDINKDSLDDLIVSAPLFNDVATGYDQGRVFVYINDPKDTKLQKWVSASYHPQMLSGSNAKGARFGMSVAVVGDVDLNGYDDIAVGAPFEEIYSTGSGTVYIYFGSSEGLIETRKQRILGKSVSPSLKTFGGFITSGAGSKNDVDGNGYSDVIISSPASDNIIILRARPIVKVQTQITLKPPSIDLINCLSSSVAPCTSAAVCLSAFYTDNRTLTAPARINVTMKLDSLLGNPVNKRLVFAGTTSSTHTMVYNVTSRSSCFTLPVRLNSAYFASTLSTGDFGIAARVEASYDYTVNHKNTIMSAVIDPLLPRVTTADWTLEKQCSEINGTRCSHDLKIETVFSVPSLGEPLILRSNPGIISINVTITNLGPDHSYFTRVNVVGNLDLNKVFGACVTVAPTNTPNDTTVSFKSENSILQGFMLKGEKCSFILEYSLLSLTKRAMVQDILLKGSVYSGVNDTASVIDPIMHNNQFEFRKKVLYTATIVQTSNSAPNAMFYNRTDSTTASIYNISNSYLGDSKYAVFHNHSVYNRGPNLVAKASLVFTWPRQTKEGLPLLYLYNFQCLPSTLCHCETLNKVNEYGLAINNETGLKNISIDFNSNTTLPSETTCNDVECDSIMCSVEQLGKFSTVVVTSSFKVWLPTLTQNKLVTKLSSKTSLNVTDSPIYNAAATTTKATTVVSEYVPPVPVYESEINLGLLIGAIVGGICLLVAIVLIMWKAGFFESKYAKLRYDAEHTDDNEITMHSNIE

Ci_a7 (Refined from ci0100152615)

MRTSLREMYICASMIVILCASMISAQNIETREFHSLSPIKSDSAVIHFGASVVIKTSPMKSIAYVGAPADGNKNGSVYKCSFSGKSYGNTSCSKDISFDNNAAIGMSIGADSSFSNLYVNMWEPTHNKLPDHTAAKTNGRGLRCPKIILIADIMFVLDDSSSVDDTAFRSALNWIIQVVSYFSSYIDSGDLRVGVYGFSNDDHRSGIRIEQIGELLNVKSTGAGTYISHAIKETVKVFEANGRKGISKEIILLTDGGASDWWLLKGEADTARDKGIVLVSVGVGTSVNNDQLLAIAGNKSRVFQATDYNTLDEVVNNVVDKTLTARTNLPNANLGAHLILQRNNKMYVGAVGSYDRAGTVVDYSASAGSVSTQYTAVLNETDYAALAGSKNHSNIANSYLGISYSVTSGLFFGGNIEYVASGAPAHESKGMVVIYKSDNVTGFPPKYLLFLHPSETSGLWQLGAYFGHSVCAVDINKDSFDDLIVSAPLFNDMNGYDQGRVFVYINNKEGIHGLLQTSESYRPQSLSGSNAKGARFGMSVAVVGDVDLNGYDDIAVGAPSEGGTSLDTGVVYVYYVSFQGISETRKQRIVGKSVISSLRNFGNVVTPSSSFSNTDVDNNGYSDIVISASSSDRIIILRARPIVKVRTQITLNPPSIDLINCLSNSSAPCTSVTVCLTAFYTGDQVLTSPARMNVTVDLDSKFGNPASKRLVHSGTTFPSKTFIFNATSVNSCVDLQVHLSSSHFTSVSAKQETLDPPQVKMTYDYIDSHKNTIMSNVLDPVTSKVYTADWSFHKKCSGLNGSKCSHDLKVETTYSVQQLGQPLILGSNPGLISINVTITNLGPDHSYFTRVNVTGNLDLNKVTGSCVTLLPVQHTNTTTVSYKASVSSVLDVMVKGVSCNFLLEYSLLSLTQTSETKEILLNGIVYSNSVNSNLTFDPDMTNNVFEYRKTVLYATTLRQTSESQQSAIFYNRVVSKEAKIHNLSNLHLGEKANFITHKHMVDNRGPNIVAKASLVFTWPRQSKEGLPLLYLYHFQCLPLKTCNCSGLNKVNEYEFAINNGTGASLFTLNYDSKATFPAQTTCEHTNCDSITCSINHLKTFSNVTIISSFKIWIPSLSQVYIVTTFSSHIRFNTIDSPVYPPSNKTTKATTKVSAYVAPTPPPDPNQVTGAVVGAVVGGCSLLIVTVFVMWKAGFFESKYAKMRQEAEQEEAESKEKGAQEKNK

Ci_a8 (Refined from ci0100130149)

MDSIKNLKYLLASIVIFSCVTDTQAQNIETRAYHSLSPIKSDSAVIHFGASVVIKTSASRSQTFVGAPLYDRKNGSLYKCTFDGKTNGSTQCLREAAFDKSGAVGISISKDNNGESIYVSVRKSTRHQLSKISIAKTDDRSLTCPIGKLTADIIFVVDESGSVDTVEYLSSLNWMKQVISSFRSYIDTGDVAVGVIGFSYDDDIDTGVRIPTIYKPLHLSGQIDNMVNIRSTLGLTYIGYAINLAITEFNSNGRKSIPREMILLTDGRATDPANVQPAAEKARANGIVTVSVGVGSGIIEDQLLTIAGAASRVFKATDYDNLASVVEGVKSTIQDTASVKLEGQITNTTSLLAECQLGISSHVTKNNKMYVGAVGSYDRAGTVVDYSASAGSISTHYTSVLNITEYSNIASASGSNASLSDTYLGYSVTSGNFLGGNVNYVASGAPKYNSKGMVAIYESNNVIGSSTNFLLLHPSETSGLWQLGAYFGHSVCAVDINKDSLDDLIVSAPLFSDVTTGYDQGRVFVFINNPKDVSLQKWVSRSYRPQSLSGSNAKGARFGTSVAVVGDVDLNGYDDIAVGAPFEEISSTGSGAVYIYYGSSKGLSETRKQCILGKSVTSLLYTFGAFITMGAQNDVDGNGYSDMIISAPSSDRIIILRARPIVKVRTQITLNPPSIDLINCLSNSSAPCTSVTVCLTAFYTDETPLSSPARVNVTMKLDSLLGNQGNKRLVFAGTTSSTHIKVYNVTSLRVCYTLPVQLNPAYFESTLSTGDFGIVSRLEVSYDYTASHKNTIMSAMLDPWSPRVTTADWSFQKKCSEMNGTRCSHDLKIETIYSVLPFGEPLVLSSNPGVVTITATITNLGPDHSFFTTVDVIANLDLNKVSGECVNVAPLNTPNVTTLSFKSKKSTLQGFMGKNEKCTFIMKYSLLSLTKKAVVKEIILKGAVHWNVNDTINAIDPVLQNNQFEFRKKVLYKAAIVHTSDSAPNSMFYNRTVSSTATVHNISNDYLGDPTYALFHSHSVYNRGPNLVAKASLVFTWPRQTKEGLPLLYLYHFQCLPLTLCHCATMGKVNEYGLAINNGTGLKNISIDFNSNTVLPTDTTCNDVKCDSITCTVNQLGKFSTVVVTSSFKVWLPTLAQRKLVTKISSTTSFNIAYSPIYKAGVTTSKATTVVSKYLPPVPPIDQNKLDIGPLIGAIVGGIVLLVVIMLIMWKAGFFESKYAKMQKDAQRETDDLDKTIEVDVNEX

Ci_a9 (Refined from ci0100136874)

NLKESITKKRFTISNKYKLSVMELTFLFSIAMLFSCTMGYNLEGRLPLYKSGGTNSTFGFSVALHKLSIDPGSSLEKSLLVVGSPTAEALPSQTNAVKPGGIYHCPVSTNSEDCTRIAMDLGESSRTNNKTGQWLGVSLKSQGAGKYLITCAHRHSMRLYSRVNEVERLCGKCFLLNGKYTKNEDLNHTCYSLCKRAPDHDGADYAQAGTSAAFAGDNEYLIGGPGMWSWTGGMFDIQMADLEATEALDIFTSSTAMYETLGCYKNELDRGKVKCDKDSYLGFSVASSNAIKPGETTYVAGAPRGNYTGAIVMYRKISNLAPILTDTILGEFIGSGFGFSVATIDVNGDGLDDLLVGAPQYYEYSNEGKHGGAVYVYINQRGVAFSQITPKKLLGSLDSFFGYSIASVGDIDQDGYIDFAVGAPQEKPNGKVYIFRGSADGNVKQSQVFNGANFTDIKPDVKGFGVSLSGAVDIDNNKYPDLLVGTLSDYVVLLRSRPVITIESSLSASPTSLDLRNPNCTLASKTYSCFELQFCFRYNARHKEYTEILPIHYSITLDSELQKERKAARVAFASPQGPSLMSDVANVPRAGNYFRNSQYIYSMKGSIWDTLSTSYLNVLIVEINGVKRERPDQPVFNMINDPIMDADFPHARTSVVNLANNCGDDGCQSNLKLRGSIPPEVVVGKLVGRDSKMLLSLNVTNDGEEAHQAVVSAKLPAWVYYDNYTVTRSSGAEIVCTTSDIGGSTFVVCTLGNPYVEGSADTIDILLDVQQLTADTKQIVIEVTGSTTSINPEIPPTPLYSDVIIQMMLSLSGYGKPQQVRYVNAPIKGESAIHYTNEIGPFVEYTFTVKNDGKYHXLQLANGVRLIVDFPIEINNGKWLMYLVSAHVRSGGLQIVGECEQQYQNTLHFKLPVTASVSAARRTKREVTSSLYEPRRLSSTQSGSNFVTLDCHGDTARCVKIGCTLDPIQPGSTTSVNLQARLWNNTFLEEFVDIDLVNVAASATVSNIDSNVRITGSPTHEALTTVDHAYFEVSVKSSFPWWYILIGILIALIIYIIIIYLLIKCGFFKRKKYPRNQHHAQDDNGAAVAEEEKEPTNRETAT

Ci_a10 (Refined from ci0100144349)

APNHPGFSLDTGRLYGRFINYVAGAPRANDTGSVVVFEKEEDAKVLTMKEILQGENLASSFGYDVKVIDITGDGREDLLVGAPQYYDKEERYGGAVYIYVNKGLSKIEIRPIMMTHRLYGDVDSSFGNSISTLGDINMDGTNDVAIGAPGADKGSGAVYIFHGNPDPNLGVYEEPAQVRLIDFNVTGFGYSLDGGLDMDMNEYPDLLVGSLSEVAVMYRSRPIINVYASIKASVKKINLNPDXQMNTRFNAIVCMNYTSTPISFDEAVNMDFTARIDSERLENDLRSRGTFSPTNLQQNSITQMLKLKPQSSRQSVCVTLPVYISHEVQDKLSPIQLDLTFNVLEEAIPPTTNVMIPLNKYPVLNNQVPRHDVTNVQISKDCGPDEICRSQIMMSANYVVKYKGSEDWVTLKPGNLGVPTLLLGTEEEIGLRVNIANPTPGEDAHQAKLRMYLPPILNYVGLDKVNATVSGRCTTVGNNASFVECSLGNPLETDSRITAIVKLENDERLYKTNGFVIQLQVLQPGHDDNVTYPVNVRVQAKIMIDGYTNVDQVRFGGEIVGESAVNVPQDAGTYITHGYEVINAGTSKIEDVYVNITWPQVIHNGKWLLYLIDTQVTGSGATNETICHVPTKQTNPLNLKYRSSGATRRKREAGGPENKVSPVVSPPTSSHKTLKCDEAADSAKCVFFLCYLGTLEKLRKVDINLRAVVWNSTFLEEYNGIGEIRVFSKAEVVVSGTNIQYSPTSVVTDEVATSVLSEEVIAPQQEVKLWIIVVATLAGVILLVIIVLLLWRCGFFKRRRD

Ci_a11 (Refined from ci0100154687)

MCAPQFAQKSVVTRSSGKSIYYDLVGICYFTTDFSADNLVTYRYSPCSSDLGSHYADLHSYCQAGFSAAFTKDNRKLVLGAIGSYYNQGSIMLVKDLLQNISSLPYHTAEFKPDVPSGWGEVDYYRHTYDSNYMGYSVATGITGSNLPLLVTSAPRRLGYNLLGSVIVYDETMQAPLANFTGEQIGEYFGEGLEVVDLNNDGLDDIIIGSPLYSDVKQKVPEIGRIYVYYQETERRLEFSPPTIINGNSSMARFGRTIVSIGDVNGDGYNDILVSAPYERNGTEDSEEKYGALYLFNGGVGGVRSYPSQVIHGLKLENTPNFTPGDKIRGLGYGMKGGKDTDGNGYPDVVVGAYLSDKVVVIKARPVVKLLVIQTITPNKIDLETLSCDLNPTTKSACFYVETCFSYSGKTLPDTVNMSYSYDVDSGKEEREKRSYLLDDSPRNLTLRAGDQQQCVHETVFMKKDVRDKQTEIAVTVNYWLQENHLPTEPVLDVLAGTSSTTRADIFKDCGPDEICIPDLVVNAKLSPDTVQVGKYSEINVKASVWNNGENAYLTTMVVNYPQYVTFIGLQEDKVDGRVISCVDLNPFLLCEVANPLKVDTRIDLVIQFGVNELQGDVDVLPLLLYANCTNEQNNQSPLFHTMIHVEVVANVKFYNVSTPSLIRLDKESENAALNQSASKPLTHTYEITNAGPAVISKAEISLLWPLSVNGDSKDLLLPLLEVQHSGPVICHYSHIADAICNKNTFSSFSSLQNCHTDPQNCYEMTCDILEMQPKTDILIELKTELQLAPVLRTSMDTVITSSMQFRITEFPYLINPGPGPTTLSEVNTYAEYPQVPESSTIEWWIIAIAVAAGVLFLLLIILLLWKCGFFKRMTHPQSEEDKAQQQKL

Ci_b1 (Refined from ci0100141446)

SNVGESAVGKATQIRPQIINSRIRPGDPLNIELSFKQAEDYPVDLYYLLDLSKSMENDLNSLRALGRELGTSMQNITRDFRLGFGSFIDKTVMPYISTVPAKIRNPCNDKAPCVPTYSFHNDLPLTPEIDAFVNSVNNVTHSSNLDNPEGGLDAMMQAIVCKEKINWRKDATHLLVYSTDASFHYAGDGKLGGIVLPNDGNCYLDDNGHYYNANAMDYPSIGHLVRKITSHNIQPIFAVTTSVIQTYTNLQKMIPKSVVGELSGDSSNIIQLIQNAYNDLKGQVLLEIRAPPEVTVSSQTAHCQNQTIPGMQCEGVKLGDIVNFTFTLSTEKCLKSPVQVLVSPYGYNEVVTINVESHCDCQCEETQAPTTNCSGHGVYECGSCVCETGFTGLDCSCDQKDVLGIESYLANCTDPATGVVCNSGGECQCGSCICKQYANKKIDGKYCECDNTTCDRAGARVCNGFGKCNCGVCECQNGWKGKACDCTLDQTECYDLSPDAVDSSKPCNGNGECECGQCVCNSRGGAKFRGQYCKTKPLVICDIHKDCIQCKAWKTGNYNTTECEKQCTKYNVTKMSKEYQYSGYTNECRFTDLTDDCNYQATFEEIDGIIMVEVEPDKTCTTYANPVYIIIGIIAAIVGIGLAILLIWKLLTSIKDAREYKNFQKESQNPKWQGGENPIFKKATSTFKNPMYSGGKTAGN

Ci_b2 (Refined from ci0100143908)

MERIKLFMLFAVLVVYSTQVSGQSTTDIDKLCEDTAITSCDQCIRKHPQCAWCSRTSDNSNIKRCRSYTKTLQASACATADITMPRSDVIFQKNLPFSSRDDINVVQLRPQNININLRKGDTVKFNVTFRKVKDYPVDLYYVMDLSNSMKDDLAELQKLGARLAEEIRQNVTSDINMGFGTFVDKVMMPFTSTVPDQLKNPCVKADEFCSAPFGFRHQQPISGDLTAFKESVQNTNISGNIDSPEGGFDALMQIAVCGNKIGWRESASHLVVFTTDASFHTALDGKLAAILDANDMQCHLTKVNSSVDSNVYVYDKSKELDYPSIGQLRAAFVANKIQPIFAVTKEVRSLYDGLKSLIPNSFVDELANDSNNIISIIKTAYNVSHRCVYYVTEYPHVTTVIFINTENPNSLETNSLSCDQIKIGSEVTYEFTISASSCPATLPSQPMTFTSSSLQEFVKFNFNFLCDCNCSSMAEVDSPSCSTNGSLVCGACLCNDGHEGSRCQCTQSTIGLSLNAQCKRSDTADICERNGRCVCGVCDCDENYHGKYCQCTNTGCPVTDGNVCGKLCVGGKCENCYNQPKCNCTSKNGIPYTLDNVGSCTCHESECIDQRSNNTMVCSGNGNCSCSSCICDPKYDGIYCQHCNSPSCKTITAECSSHEACAICNANDLCQVQCKDVTFQTVKAIPDCSVCTANTQSTGQCKVSYNIVWKKSLRNYLVLIKEFDQAIDCPKPINPLVIVLPIVAGIVILGLIALVAWKVYQTWRDKREWKVFENEMKKSKWTKGQNPIFEEASTRFENPTFHGT

Ci_b3 (Refined from ci0100153278)

MVCNDVYGLIAWFILLMVCFTWTVESQTQNAVTDPCTAATTCADCIKINPMCTWCADNKTTPGTRRVRCKLLINNQRDCLTGFIENPKSISAPTKNLPFSNVTSEVFENPATANMTILQLKPQAINVKLRPTGQMETVNITFRKVVDYPLDLYYVMDLSNSMSDDLATLKGLGDSLAADIRNVTKNVRLGFGTFVDKVVMPFASTIPDQLRDPCLKIANETCAPVFGFRHQLGITADGALFREAVNSTLISGNIDSPEGGFDALMQITVCQNIIQWRDEALRVILYTSDASPHIALDGKLAAILEANDMRCHLEADAIVYSKSKNMDYPSIGQLKHVFDQYKVQPIFAVTESVFDLYTGLPQIIDNSFVGKLRSDSKNVVQVISDSYNRLKSLVQLTRPTKPSNIDMTYRVLCPGATWVDNSLQCDNIEIGDEITFQFQLTAQTCPTNTAQKDSIAITSSSLKDEVAISIEYICDCECSNAPLLNHSQCSDSGSLQCGVCQCNSGTDGSQCQCTASEVALNRNCSIDTDCTGGNGYCECGKCICNVGFFGRLCECRSTGADKGVCNNCNNDGRCICNSGGGYFLNNITNTCICNLKDCADVGGTLCNGRGTCECNTCECTDTRLYSGATCQTCNLPECTGIDGTCSGTTIRACVECIYDNQRLKKVNLYPPHDTLIAPNQKSLIDCPNYEPYRKQSPTKLPRDLLIITFLVPDCPQPINPWLIVGPVVGGIVLIGLIILIVWKIFQTIKDKVEYEQFLEDSKNKTWSKGNNPLYSKASVRVVNPAFE

Ci_b4 (Refined from ci0100131678)

MFALFIQAESDCKEVCLDKDIRTRVLSSVAQCDKCKESYTDACKHLIDTTQYIVGLANHWRRVVDQYDRVTCGKNIENKLRQTEMGGVSSGSQEK*SAQLSCKQCITTNPRCTWCNDNRVPTAIRKNCKLDRYIENNQTCSVGVNSPQTLINKTINEEFTAENKTNVTHPIQLRPQEISLKLRIGQPTKVNITFHKLIDFPLDLYYVMDLSLSMRDDLAQLKILGSSLIDVLRNVTVNTRLGFGTFVDKVIAPFASDNKYIVKDACDSVSTTCVEPFGFHHQLKMSSDTTKFKQAVEETIISTNIDEPEGGFDALMQIAVCQDIIGWRKESLKVVLFTSDDSPHIALDGKLVQILKPNDMKCHMDLIKQIWEYTESKTQDYPSLGQLKHVLEQNKVQTIFAVTQNMRSMYEDIHSLLPNAHIATLNTDSSNIQDIISSSYDKIRSRFAFNKPTAVKGLDIKHRVLCHGTDTWSTSLLCENTKQGQEVIIELEITANECPSSGSQTDVIKVTSDQISDVVTIKVDYHCKCSCEDEIASNNRSLCSFNGDFLCGMCNCDSGHTGSYCQCEATTTINMNVNCTDPKSPNGMCNNNGMCDCGECICSPSYNGTFCECSSTGCPSASGSMCGGSDKGRCENCYGNKQCVCNTNDGWYLDQSGECTCNNRSCMAAGSNVTCSGHGTCDCSTCTCSENIVIHN

Ci_b5 (Refined from ci0100143050)

SSSSSSSTSSSVCVQAKVPEDCIKVNPNCQWCAQEDFSGQRCNIQPILEEQGCSDLQIIESSVAMDAANEANRFDHQSASVSVTSLCRDARIRVGGNVNINFRIREPMVYPVDFYYLMDVSYSMLDDLKSVQTLAQQLVATLQELTQGGTGSVKLGFGKFVDKVQSPMTQMTPYKLQHPYGSSTDAPFLFRNVIDLTSDVDKFEQVLSKQNVSGNLDPPEGALDAMLQVVKCKELIGWRGDALRLLMVATESAFHFAGDGSGYLAGISRANDGRCHTGGDGIYVGAEEQDYPTVNQVVRAVEENSITPIFAIGKTYADMYKYVSEDVFRGSTWGILKKDSSNVVDLVRRAYLDITGKQEIVTSNPDDSVLCPWYHVTRPGGAMEQLSVDGTLVGGLESGSKVYYNMSLYANRGACAGATSCPVGDVIVKTRQYDDKLTIATSVVSAFGCAATVIVNSPSCSGNGNFSCGICRCLPAWGGSSCSIPVEGKIGCLPLDGGAKCSGRGKCLNNRCQCDPGYRHGKSKIFGDFCECDECPVRAGLVCSGNGRAVTGTGCSCDCVCDAGWEGSDCACASNTTTCMGLGGEICSGFGQCVCGACVCDVTSGYSGPTCSDCVQNCPSCAAFKDCIQCTMHQSGALADSCVTSCNDEVIEDIDEASGEVLCAALDKIDNCKFYYSYKPSGSGMVFKAEVEKRCKPEYSLLIILWCILLFLLIGLILLCCWRCCVYVIDKEESKNYKLSNI
